# Knowledge of women in family planning and future desire to use contraception: a cross sectional survey in Urban Cameroon

**DOI:** 10.1186/s13104-016-2155-7

**Published:** 2016-07-18

**Authors:** Atem Bethel Ajong, Philip Nana Njotang, Bruno Kenfack, Martin Ndinakie Yakum, Enow Robinson Mbu

**Affiliations:** Department of Obstetrics and Gynaecology, Faculty of Medicine and Biomedical Sciences, University of Yaoundé I, Yaoundé, Cameroon; Obstetrics and Gynaecology unit, Yaoundé Central Hospital, Yaounde, Cameroon; Department of Biomedical Sciences, Faculty of Science, University of Dschang, Dschang, Cameroon; Meilleur Accès aux soins de Santé, Yaoundé, Cameroon; Directorate of Family Health, Ministry of Public Health, Yaoundé, Cameroon; Dschang District Hospital, Dschang, Cameroon

**Keywords:** Knowledge, Family planning, Contraception, Future desire, Cameroon

## Abstract

**Background:**

The rate of modern contraceptive use will be on an increase and maternal mortality on a decrease if women had a good knowledge on family planning and its methods. This survey was designed to evaluate the knowledge and determine the future desires to use contraception among women in Urban Cameroon.

**Methods:**

We conducted a cross sectional community based survey from March 2015 to April 2015 targeting women of childbearing age in the Biyem-Assi Health District. Participants were included using a multistep cluster sampling and the data collected face to face by well-trained surveyors using a pretested and validated questionnaire. The data were then analysed using the statistical software Epi-Info version 3.5.4. Proportions and their 95 % confidence intervals were calculated and in a multiple logistic regression model with threshold of significance set at p value ≤0.05, the odds ratio was used as the measure of association between selected covariates and future desire to use contraception.

**Results:**

Among the 712 women included in the survey, the mean age was 27.5 ± 6.5 years. A good proportion (95.6 %) identified contraception as used to prevent unwanted pregnancy and this showed an increasing trend with increasing level of education. Also, 77.5 % thought that contraception should be used by all sexually active women. The most cited contraceptive methods were; condom 689 (96.8 %), oral pills 507 (71.2 %), and implants 390 (54.8 %). Their main sources of information were the health personnel (47.7 %) and the school (23.6 %). It was estimated that 31.0 [25.5–37.0] % of current contraceptive non-users had no desire of adopting a contraceptive method in the future. With the level of education, age, and marital status controlled, the number of unplanned pregnancies more than 3 (OR 0.66 [0.45–0.97], p = 0.035), and past adoption of more than 2 modern contraceptive methods (OR 0.45 [0.21–0.97], p = 0.041) were statistically significantly associated to decreased desire to adopt contraception in the future. The level of knowledge showed an association though not statistically significant with future desire to use contraception (OR 0.80 [0.47–1.37], p = 0.061).

**Conclusion:**

The knowledge of women of childbearing in the Biyem-Assi Health District was relatively high but still unsatisfactory. The proportion of contraceptive non users who have no desire of adopting any contraceptive method in future is still unacceptably high. Policy makers should improve on their strategies while empowering the health personnel and working in collaboration with the education ministries.

## Background and rationale

The rate of use of family planning services in the developing world and more particularly in Cameroon remains unsatisfactory [1, 2]. The number of women with an unsatisfied potential demand for contraception is still very high in Cameroon [3]. Meeting targets of the Sustainable Development Goals in maternal and neonatal health requires a good and consistent use of family planning services. With abortion not legalised in Cameroon, the effective use of family planning methods remains the mainstay in the fight against the already high rates of induced abortion and its complications [4–7].

The adoption of any contraceptive method requires prior knowledge of the method. A good knowledge of the use of family planning methods and their benefits/side effects depends on the effectiveness of the counselling and sensitization of the risk population [2, 4, 6, 8]. Also, the perception of family planning by women is dependent on good knowledge and has a great impact on their attitudes and practices [7, 9, 10].

A recent survey (2015) in Yaoundé–Cameroon showed that 20.4 % of women in a union had an unmet need for family planning and that the potential demand for family planning was just at 70.6 % [3]. According to the national demographic and health survey in 2011, with a contraceptive prevalence of 24, 94 % of women of childbearing age in Cameroon had knowledge of at least one modern contraceptive method. Also, about 46 % of contraceptive non-users in 2011 had no intention of adopting any method in the future [1].

Given that a good knowledge influences perceptions and will improve on the future desires to use contraception. Policy makers need to stress on the need for sensitizers and family planning providers to remain very explicit in their interventions. A good knowledge and perception is indispensable for the fight against the unsatisfactory potential demand of family planning services and therefore its consequences. This survey was designed to evaluate the knowledge and the future desire to adopt family planning methods in a sample of women in urban Cameroon among which unmet need for family planning had been reported high [3].

## Methods

We conducted a cross sectional community based survey in the Biyem-Assi Health District (the largest health district in Yaoundé with a high prevalence of unmet need for family planning) [3] targeting sexually active women of childbearing age. Not included were visitors to the district, and women who were mentally incapacitated at the time of our survey. The Biyem-Assi Health District is an urban health district with a cosmopolitan population dispersed over a total of four health areas (the Mendong, Melen, Biyem-Assi and Mvog-Betsi). The minimum sample size of the study was calculated using the following parameters: the expected proportion of non-contraceptive users with future desire to use contraception (46 % according to the national demographic and health survey data 2011) [3], the absolute precision required on either sides of the proportion (0.05), threshold of error at 5 % and a cluster effect of 2. The minimum required sample size for the survey was estimated at 680 participants and 712 were included in the survey using a cluster multistep sampling.

In this process, all four health areas were included in the survey and the whole district divided into a total of 70 geographical clusters from which 50 were randomly selected with replacement. In a selected cluster, a road junction was randomly selected from all major junctions and a street selected by tossing a plastic bottle. In a selected street, all households on the left hand side of the interviewer were included in a successive manner. Prior to data collection, a two-day training session was organised by the principal investigator to enlighten the surveyors (of both gender) on the consenting and data collection procedure. All eligible and consenting participants within a selected house hold were included in the survey. Data were collected using a pretested and validated questionnaire administered face to face by well-trained surveyors. Data collected included evaluation of the knowledge of participants on family planning and their intentions to use contraception in the future. The data were collected from March 2015 to April 2015.

### Data analysis

The data from filled and validated questionnaire were then entered into a predesigned data capturing sheet and analysed using the statistical software Epi-Info version 3.5.4. Major analysis included calculation of proportions (current contraceptive users, future desire to use, the different knowledge indicators etc.) and their 95 % confidence intervals where relevant for qualitative variables and means with standard deviations for quantitative variables. With potential confounders like age, level of education, marital status, and religion checked in a multiple logistic regression model, the odds ratio was used as the measure of association between selected covariates (knowledge, number of methods used in the past and number of unplanned pregnancies) and the outcome of interest (future contraceptive use). All with a statistical significant threshold set at p value ≤0.05). The knowledge variable was divided to level of knowledge below or above average depending on the number of right answers given to the set of questions to evaluate the knowledge, that is above or below 50 % of correct answers. The number of modern methods adopted in the past was divided into the number of modern methods adopted in the past above two or less than or equal to two while the number of unplanned pregnancies was divided into above three and less than or equal to 3 years. The results were presented on tables, figures and some written out.

## Results and discussion

**Fig. 1:**
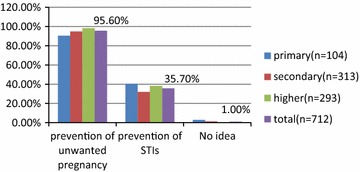
Uses of contraception or family planning as indicated by the participants

Among the 712 women included in the survey, the mean age of the participants was 27.5 ± 6.5 years and the most represented age group range was 20–29 years. Most (60 %) were in a union, 96.7 % Christians and about 8 in every 10 had acquired at least a secondary education. All the women interviewed had heard of contraception in their lifetime. Figure 1 shows the uses of contraception or family planning as indicated by the participants. A good proportion (95.6 %) identified contraception as used to prevent unwanted pregnancies and this showed an increasing trend with increasing level of education. A survey carried out in 2010 in the Mbouda Health District in West Region of Cameroon found out that 96 % of the sample surveyed had heard of family planning and about 80.3 % identified it as used to prevent unwanted pregnancies [2]. This slight difference in the results could be explained by the semi-urban setting of the Mbouda survey and the increasing sensitization over the years on family planning. Among refugees in Cameroon, it was found that only about 80.7 % had ever heard of contraception [11]. This is low compared to our findings but however normal given the life conditions of the refugees and the little exposure to information on health and more precisely reproductive health [11]. Also 35.7 % said some family planning methods could also be used to prevent sexually transmitted infections (see Fig. 1).

Of the 712 participants, 552 (77.5 %) thought that contraception should be used by all sexually active women while 35 (4.9 %) thought only women with at least one child should use contraception. Unplanned pregnancy is a problem among all sexually active women, therefore contraception should be adopted by any woman who is sexually active and not planning to get pregnant any time soon. A relatively high proportion thinks only women with at least a child should adopt contraception. This could be explained by the beliefs and misinformation on some reversible family planning methods associated to risk of infertility.

**Fig. 2:**
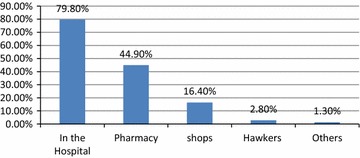
Sources of modern contraceptive methods

Figure 2 shows the identified sources of modern contraceptive methods. The most mentioned sources of modern contraceptive methods were the hospital 568 (79.8 %) and the pharmacy 320 (44.9 %). These are the correct sources of modern contraceptive methods. A survey among students and staff in Delta state Nigeria also identified the pharmacy and the health centre as major places where family planning methods could be gotten [12].

Table 1 presents the knowledge of the participants on the different family planning methods. The most cited contraceptive methods were; condom 689 (96.8 %), oral pills 507 (71.2 %), implants 390 (54.8 %), injectable 325 (45.6 %), and IUD 311 (43.7 %). The trend is not very different from that reported in a 2010 survey in Mbouda Health District during which 96 % knew the condom, 76.2 % the injectable, 75.2 % the oral pills, 56.4 % the implant and 39.6 % the IUD [2]. However the differences could be explained by the differences in the methods adopted in questionnaire administration and also by the fact that Yaounde and Mbouda are found in regions with clearly different socio-cultural beliefs. In addition, the organisation of the family planning services in these two settings is not identical. The condom remains the most cited method of contraception among women in Cameroon. This was also true according to the 2011 Demographic and Health Survey in Cameroon [1]. This is because of the dual function of the condom which has made it highly publicised over the media and campaigns on the fight against HIV/AIDS. Also, 701 (98.5 %) of the participants cited at least 01 contraceptive method with 699 (98.2 %) and 518 (72.7 %) citing at least one and three modern contraceptive methods respectively (see Table 1). This is proof of a relatively good knowledge of these women on the different contraceptive methods. This result is in line with that reported in the 2011 Demographic and Health Survey in which 94 % of women of childbearing age were knowledgeable of at least one modern contraceptive method [1]. The mild difference observed in the results may be explained by the highly urbanised nature of the Biyem-Assi Health District which could mean easy access to information on family planning or could be due to sample variation. In addition, the level of education of women of childbearing age in the Biyem-Assi Health District was relatively high with 85.4 % having acquired at least a secondary education. Out of the 699 women who cited at least one modern contraceptive method, 674 (96.4 %) identified the condom as used to prevent STIs. This is very important given that the use of condoms among sexually active women is the mainstay in the effective fight against sexually transmitted infections and the HIV/AIDS pandemic.

**Table 1:** Knowledge of different methods of contraception stratified by level education of participants

Indicator	Level of education (frequency in %)			Total n (%)
	Primary, n = 104	Secondary, n = 313	Higher, n = 293	
Can you cite the different contraceptive methods you know? (n = 712)				
Condoms	97 (93.3)	299 (95.5)	291 (99.3)	689 (96.8)
Diaphragms	5 (4.8)	12 (3.8)	47 (16.0)	64 (9.0)
Cervical caps	2 (1.9)	2 (0.6)	16 (5.5)	20 (2.8)
IUD	36 (34.6)	120 (38.3)	154 (52.6)	311 (43.7)
Spermicides	1 (1.0)	15 (4.8)	24 (8.2)	40 (5.6)
Implants	48 (46.2)	172 (55.0)	169 (57.7)	390 (54.8)
Oral pills	60 (57.7)	218 (69.6)	227 (77.5)	507 (71.2)
Injectable	50 (48.1)	151 (48.2)	123 (42.0)	325 (45.6)
LAM	1 (1.0)	19 (6.1)	17 (5.8)	37 (5.2)
Coitus interruptus	6 (5.8)	35 (11.2)	54 (18.4)	96 (13.5)
Cycle	15 (14.4)	46 (14.7)	53 (18.1)	115 (16.2)
Others	1 (1.0)	6 (1.9)	6 (2.0)	14 (2.0)
No idea	3 (2.9)	5 (1.6)	1 (0.3)	9 (1.3)
Number of cited modern methods n = 712				
0	4 (3.8)	8 (2.6)	1 (0.3)	13 (1.8)
1	22 (21.2)	27 (8.6)	21 (7.2)	70 (9.8)
2	15 (14.4)	59 (18.8)	37 (12.6)	111 (15.6)
3	20 (19.2)	86 (27.5)	74 (25.3)	181 (25.4)
4	29 (27.9)	89 (28.4)	93 (31.7)	212 (29.8)
5	13 (12.5)	38 (12.1)	50 (17.1)	101 (14.2)
6	1 (1.0)	3 (1.0)	9 (3.1)	13 (1.8)
7	0 (0.0)	2 (0.6)	6 (2.0)	8 (1.1)
8	0 (0.0)	1 (0.3)	2 (0.7)	3 (0.4)

*IUD* intra-uterine device, *LAM* lactation and amenorrhoea method

Table 2 shows the sources of information of the participants on family planning. Of the 709 participants who responded to the question on the main source of information on contraception, 338 (47.7 %) said their main source of information was a health personnel, 167 (23.6 %) from school, 112 (15.8 %) from family, relations and friends, and the remaining 92 (13.0 %) said it was the media. It is clear from here that to meet the targeted population, the health personnel need to be empowered in this domain. A survey among secondary school girls in Tanzania identified the school as a major source of information on contraception [13]. Knowledge could also be increased by making sure that every student leaving secondary education in Cameroon acquires at least a course in family planning. Comparing this to their main source of information, 629/702 (89.6 %) said the health personnel was the best source of information on contraception. Though a good proportion correctly identifies the health personnel as the best source of information, more is still to be done to understand the limits to access of this information on family planning and contraception.

**Table 2:** Sources of information on contraception

n (%)	
Main source of information n = 709	
School	167 (23.6)
Family, relations and friends	112 (15.8)
Health personnel	338 (47.7)
Media	92 (13.0)
According to you, what is the best source of information about contraception? n = 702	
Family, relations, and friends	20 (2.9)
Health personnel	629 (89.6)
Media	36 (5.1)
School	16 (2.3)
Others	1 (0.1)

Out of 708 participants who responded to contraceptive use, 61.7[58.0–65.3] % were currently using at least one contraceptive method and 91.8[89.5–93.7] % had adopted at least one contraceptive method in their lifetime. This rate of contraceptive practice is relatively high but does not give a clear picture of the contraceptive need of the population. A survey carried out in this population in the same year revealed that 16.4–24.8 % of women in a union had an unmet need for family planning [3]. During our survey, it was estimated that 31.0[25.5–37.0] % of those who were not currently using contraception had no desire of adopting a contraceptive method in the future.

This shows to an extent the magnitude of work awaiting family planning providers in convincing and dissuading this population to adopt effective contraceptive methods. However a survey carried out in this population during the same time frame revealed that discussion of family planning within the couple and husband’s approval of contraception were two major determining factors keeping unmet need for family planning high [3]. Also, the fear of side effects was the major reason of non-use of family planning among these women [3]. This finding is nonetheless, better compared to national demographic and health survey results in 2011, in which up to 46 % of contraceptive non-users had no future desire of using a modern contraceptive method in the rest of their reproductive life. This shows the positive efforts of the Cameroon government and its family planning care providers in increasing modern contraceptive practice.

With level of education, age, marital status and religion controlled, the level of knowledge showed an association though not statistically significant with future desire to use contraception (OR 0.80 [0.47–1.37], p = 0.061). The lower the knowledge on contraception and its methods, the less likely had they plans to adopt contraception in future. This is but logical given the fact that contraceptive use requires a good knowledge of its functions and methods. Many surveys have associated good knowledge to increased contraceptive use [3, 6, 8]. Though not statistically significant in our survey, it is a pointer to the need for intensified and focused sensitization in this domain. It is clear from here that if contraceptive non-users be well sensitized, we will have more of them adopting contraceptive methods in the future.

With the level of education, age, marital status and religion controlled the number of unplanned pregnancies more than 3 (OR = 0.66 [0.45–0.97], p = 0.035), and past adoption of more than 2 modern contraceptive methods (OR = 0.45 [0.21–0.97], p = 0.041) were statistically significantly associated to decreased desire to adopt contraception in the future. Women who have adopted more than two methods of contraception most often have suffered from side effects and inconveniences of different methods that made them move from one to another. When these complaints are not clearly addressed by the health provider, the women turn to withdraw from contraceptive use. Contraceptive non-use has been described to be highest among women with unintended pregnancies [14]. This relationship is clear in the light that contraceptive non-users are opened to having more unplanned pregnancies than users. This exposes the conservatism portrayed by contraceptive non-users and more specifically those with more unplanned pregnancies to avoid the adoption of modern contraceptive methods.

### Limits and strengths of the survey

This survey evaluated just the knowledge of women of childbearing age on family planning. The perceptions and attitudes of this population towards family planning were not evaluated. Given the vital role men play in the decision making, a survey is required among men. Also, the health personnel who are at the centre of the dispensation of knowledge and provision of family planning services were not evaluated in this survey. However, the level of knowledge and future desire to use contraception pictured from this survey shows the necessity of the health personnel and family planning sensitizers to improve on their strategies in order to improve on the knowledge of the population in need. This survey was well designed with adequate methodology and all put in place to check for bias. It types out the situation on the level of knowledge on family planning in an urban setting in Cameroon.

## Conclusion

The knowledge of women of childbearing in the Biyem-Assi Health District was relatively high but still unsatisfactory. A high proportion (31.0 %) of contraceptive non users had no desire of adopting any contraceptive method in future. Major sources of information on contraception are the health personnel and the school. With the level of education, age, and marital status controlled, the number of unplanned pregnancies more than 3, and past adoption of more than 2 modern contraceptive methods are statistically significantly associated to decreased desire to adopt contraception in the future. The level of knowledge showed an association though not statistically significant with future desire to use contraception. Policy makers should improve on their strategies while empowering the health personnel and working in collaboration with the education ministries to encourage teachings on family planning and its methods to at least all secondary school leavers. More surveys should be carried out in this field to evaluate the health care providers as well as study the perceptions and attitudes of this population.

## Abbreviations

HIV/AIDS: human immunodeficiency virus/acquired immuno-deficiency syndrome
IUD: intra-uterine device
STI: sexually transmitted infection
